# Fasciculation differences between ALS and non-ALS patients: an ultrasound study

**DOI:** 10.1186/s12883-021-02473-5

**Published:** 2021-11-10

**Authors:** Jingwen Liu, Yi Li, Jingwen Niu, Lei Zhang, Jing Fan, Yuzhou Guan, Liying Cui, Mingsheng Liu

**Affiliations:** Department of Neurology, Peking Union Medical College Hospital, Chinese Academy of Medical Sciences, Beijing, China

**Keywords:** Amyotrophic lateral sclerosis, Fasciculation, Ultrasonography, Peripheral neuropathy, Diagnosis

## Abstract

**Background:**

Fasciculation is an important sign for the diagnosis of amyotrophic lateral sclerosis (ALS). Our study aimed to analyze the difference in fasciculation detected with muscle ultrasonography (MUS) between ALS patients and non-ALS patients with symptoms resembling ALS.

**Methods:**

Eighty-eight ALS patients and fifty-four non-ALS (eight multifocal motor neuropathy, 32 chronic inflammatory demyelinating polyneuropathy/Charcot-Marie-Tooth, and 14 cervical spondylopathy or lumbar spondylopathy) patients were recruited. MUS was performed on 19 muscle groups in cervical, lumbosacral, bulbar, and thoracic regions for each patient. The intensity of fasciculation was divided into five grades based on firing frequency and number in the involved muscle groups.

**Results:**

The overall detection rates were 72.8% in ALS and 18% in non-ALS patients. The fasciculation grades (median [IQR]) were 2 (0–3) in ALS and 0 (0–0) in non-ALS patients (*P* < 0.001). Fasciculations were observed in four regions for ALS patients and primarily distributed in proximal limbs. Fasciculations in non-ALS patients were primarily low-grade and mostly distributed in distal limbs.

**Discussion:**

The fasciculation grade was higher in ALS than non-ALS patients. The distribution pattern of fasciculation was different between ALS and non-ALS patients.

**Conclusions:**

The fasciculation grade and distribution pattern detected with MUS could help distinguish ALS from non-ALS patients.

**Supplementary Information:**

The online version contains supplementary material available at 10.1186/s12883-021-02473-5.

## Introduction

Amyotrophic lateral sclerosis (ALS) is a neurodegenerative disease that involves motor neurons in the cerebral cortex, brainstem, and spinal cord [1]. Since the Awaji criteria [2] in 2008 emphasized the significance of fasciculation potential, fasciculation has been widely considered to be a critical biomarker for the early diagnosis of ALS [3, 4]. Electromyography (EMG) is currently the primary method for detecting fasciculation. However, EMG has certain limitations due to the small recording area, its time-consuming nature, and invasiveness [5].

Recently, the clinical application of neuromuscular ultrasound technology has reduced the time from onset to diagnosis for ALS patients [6]. The advantages of non-invasiveness and easy operation provide muscle ultrasonography (MUS) with unique advantages in the observation of fasciculations. Moreover, recent studies have shown that MUS has higher sensitivity in detecting fasciculation compared to EMG and qualitative physical assessment [7–10]. Currently, research on fasciculation under ultrasound has focused chiefly on scanning time, firing frequency, and distribution [11–14].

Our study analyzed differences in firing frequency, intensity, and distribution of fasciculation with MUS between ALS patients and non-ALS patients with symptoms resembling ALS and specifically explored the usefulness of MUS fasciculation grading and distribution patterns in the diagnosis of ALS.

## Materials and methods

### Subjects

Consecutive patients with ALS according to the Awaji criteria [2] and non-ALS patients who exhibited symptoms resembling ALS were recruited from March 2017 to May 2020. Non-ALS patients included those with peripheral neuropathy (PN) and cervical spondylosis or lumbar spondylosis (See Additional file 1). All enrolled patients were recorded with their name, gender, age, body mass index (BMI), disease duration, and region of onset. ALS patients were assessed using the ALS Functional Rating Scale-Revised (ALSFRS-R) [15]. Muscle strength was measured using the Medical Research Council (MRC) score, including bilateral assessment of the following limb muscle actions: shoulder abduction, elbow flexion, elbow extension, wrist flexion, wrist extension, finger flexion, finger extension, thumb abduction, little finger abduction, hip flexion, knee flexion, knee extension, ankle dorsal extension, ankle plantar flexion, toe dorsal extension, and toe plantar flexion. The total MRC score was 160.

This study was approved by the Ethics Committee of the Peking Union Medical College Hospital (PUMCH). All enrolled patients provided written, informed consent to be included in the study.

### Ultrasound study

MUS examination was performed using an 8–12 MHz linear array transducer (LOGIQ e; General Electric company, Wuxi, China). The initial settings were kept constant during all examinations. The gain was set to automatic mode, the depth and focus were adjusted depending on the muscle and individual patient variations. The patients were asked to relax for a minimum of 30 min before the MUS examination was initiated. The view under the probe always included more than one muscle, especially for the forearm. Therefore, in this study, the target of observation was fasciculation of muscle groups rather than specific muscles. We recorded any fasciculation detected in the area of the muscle group being assessed. MUS examination was performed in the cervical (eight muscle groups), lumbosacral (eight muscle groups), thoracic (two muscle groups) regions bilaterally and bulbar region (one muscle group) for each participant. The muscle groups that were tested are shown in Table 1.

**Table 1:** Regions and muscle groups assessed with MUS

Regions	Muscle groups	Muscles
Cervical	Proximal flexors in upper limb	Biceps brachii, brachialis
	Proximal extensors in upper limb	Triceps brachii
	Distal flexors in upper limb	Flexor carpi radialis, palmaris longus, Flexor carpi ulnaris, flexor digitorum superficialis, flexor digitorum profundus
	Distal extensors in upper limb	Extensor digitorum, extensor carpi radialis brevis, extensor carpi ulnaris, extensor pollicis longus, abductor pollicis longus
Lumbosacral	Proximal flexors in lower limb	Vastus lateralis, rectus femoris, vastus intermedius, tensor fasciae latae
	Proximal extensors in lower limb	Semitendinosus, semimembranosus, biceps femoris
	Distal flexors in lower limb	Gastrocnemius, soleus, flexor hallucis longus, flexor digitorum longus, tibialis posterior
	Distal extensors in lower limb	Tibialis anterior, extensor hallucis longus, extensor digitorum longus
Bulbar	Suprahyoid muscles	musculus digastricus, mylohyoid, geniohyoid, tongue muscle
Thoracic	Paravertebral muscles	T10 paraspinal muscle

Each muscle was imaged transversely using the B-mode. The transducer was adjusted to be perpendicular to the belly of the muscle groups, which also was the standard insertion site for the needle used for EMG assessment. This specific orientation allowed the maximal cross-sectional image of the muscles. The transducer was held in the same position for 60 s. The presence of fasciculation was recorded for each muscle group. The whole process was recorded in videos for all muscles tested. The patient kept the muscles relaxed and silent during the MUS examination.

The intensity of fasciculation was evaluated based on our defined criteria that included firing frequency and site number in the specific muscle group involved in each assessment (Table 2). The videos of MUS fasciculation grading are provided in Additional file 2. The fasciculation grade for each muscle group was recorded after each assessment. Grades 1 and 2 were defined as low-grade fasciculation. Grades 3 and 4 were defined as high-grade fasciculation. The total fasciculation score was calculated by summing the fasciculation grades of all 19 muscle groups. The highest fasciculation grade from all 19 muscle groups was used to indicate the maximum fasciculation score for each patient.

**Table 2:** The criteria for fasciculation grade

Fasciculation intensity	Definition
Grade 0	No fasciculation in the area tested
Grade 1	Fasciculation presented at ≤2 sites in the area tested, and ≤ 3 times in 10 s at any site.
Grade 2	Fasciculation presented at ≤2 sites in the area tested, and > 3 times in 10 s at least at 1 site.
Grade 3	Fasciculation presented at ≥3 sites in the area tested, and ≤ 3 times in 10 s at any site.
Grade 4	Fasciculation presented at ≥3 sites in the area tested, and > 3 times in 10 s at least at 1 site.

### Statistical analysis

The Shapiro-Wilk test was used to assess whether data exhibited a normal distribution. Non-normally distributed variables, including age-at-onset, disease duration, total MRC, BMI, ALSFRS-R, and fasciculation grade/score were expressed as medians (interquartile range, IQR), and comparisons between ALS and non-ALS patients were assessed using the Mann-Whitney U test. The χ2 test was used to assess comparisons between the frequency of categorical variables. Two-sided *P-*values were calculated for all analyses. A value of *P* < 0.05 was considered statistically significant. All statistical analyses were performed using SPSS, version 23.0. Figures were prepared using GraphPad Prism 7.00 software.

## Results

### Clinical characteristics

A total of 142 participants were recruited, including 88 ALS patients and 54 non-ALS patients. All ALS patients were followed for a minimum of 6 months and were diagnosed with probable or definite ALS according to the Awaji criteria. The non-ALS group included eight multifocal motor neuropathy (MMN) patients, 32 chronic inflammatory demyelinating polyneuropathy (CIDP)/Charcot-Marie-Tooth (CMT) patients, and 14 cervical spondylopathy or lumbar spondylopathy patients. A total of 1672 muscle groups in ALS patients and 1026 muscle groups in non-ALS patients were examined. The clinical characteristics of the patients are shown in Table 3. Significant differences in gender (*P* = 0.012), age (*P* = 0.017), and BMI (*P* = 0.030) were observed between ALS and non-ALS patients.

**Table 3:** Clinical characteristics of ALS and non-ALS patients

	ALS (***n*** = 88)	Non-ALS (***n*** = 54)	***P*** value
**Gender (male, %)**	43 (48.9%)	38 (70.4%)	**0.012**
**Age (years)**	55 (45–63)	49.5 (31.8–58.3)	**0.017**
**Disease duration (months)**	10 (7–18)	19 (5–52.5)	0.128
**Total MRC**	132.88 (117–144)	133 (114.31–148)	0.998
**BMI**	23.9 (21.17–25.45)	25.1 (22.05–27.99)	**0.030**
**ALSFRS-R**	42 (38–45)	–	
**Region of onset (n, %)**			
Bulbar	9 (10.2%)	–	
Cervical	47 (53.4%)		
Lumbosacral	28 (31.8%)		
Multiple regions	4 (4.5%)		

*P-*values with significant differences are listed in bold type

### Comparison of fasciculation between ALS and non-ALS patients

**Fig. 1:**
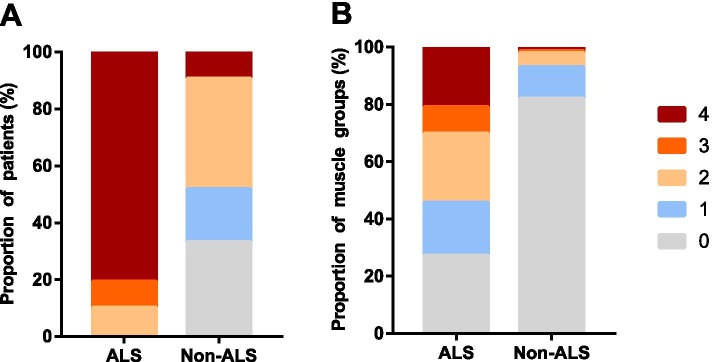
**A** Distribution of the maximum fasciculation scores in ALS and non-ALS patients. The proportion of ALS patients with the maximum fasciculation score of 4 (71 [80.7%]) was the highest, followed by 2 (9 [10.2%]) and 3 (8 [9.1%]), with no scores of 1 or 0. The proportion of non-ALS patients with the maximum fasciculation score of 2 (21 [38.9%]) was the highest, followed by 0 (18 [33.3%]), 1 (10 [18.5%]), and 4 (5 [9.3%]), with no scores of 3. **B** Distribution of the fasciculation grade in ALS and non-ALS muscle groups. In ALS muscle groups, the fasciculation of grades 0, 1, 2, 3, and 4 accounted for 27.2, 18.6, 24, 9.3, and 20.9%, respectively. In non-ALS muscle groups, the fasciculation of grades 0, 1, 2, 3, and 4 accounted for 82, 11.1, 4.8, 0.8, and 1.4%, respectively

The number of muscles with fasciculation per person (median [IQR]) was 14.5 (11–15) in ALS patients and 3 (0–5.25) in non-ALS patients (*P* < 0.001). The maximum fasciculation score was 4 (4-4) in ALS patients and 1 (0–2) in non-ALS patients (*P* < 0.001). The total fasciculation score was 34.5 (19.25–44.50) in ALS patients and 3 (0–7.25) in non-ALS patients (*P* < 0.001). The distribution of the maximum fasciculation score is shown in Fig. 1 A. The highest proportion was grade 4 in ALS patients (71 [80.7%]) and grade 2 in non-ALS patients (21 [38.9%]).

**Fig. 2:**
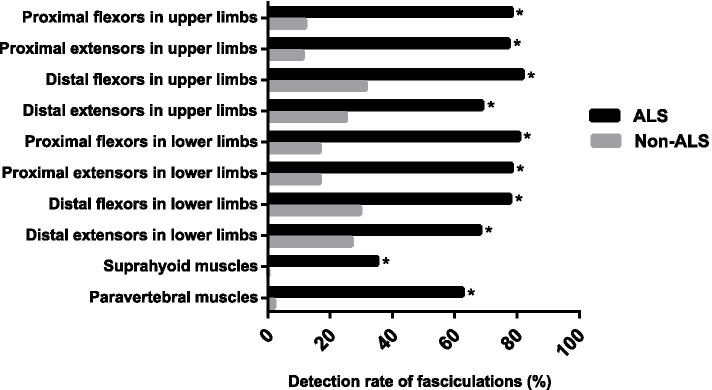
The detection rate of fasciculation in each ALS and non-ALS muscle group. The columns represent the fasciculation detection rates for proximal flexors in upper limbs (78.4% vs. 12%), the proximal extensors in upper limbs (77.3% vs. 11.1%), the distal flexors in upper limbs (81.8% vs. 31.5%), the distal extensors in upper limbs (68.8% vs. 25%), the proximal flexors in lower limbs (80.7% vs. 16.7%), the proximal extensors in lower limbs (78.4% vs. 16.7%), the distal flexors in lower limbs (77.8% vs. 29.6%), the distal extensors in lower limbs (68.2% vs. 26.9%), suprahyoid muscles (35.2% vs. 0) and paravertebral muscles (62.5% vs. 2%) in ALS and non-ALS muscle groups, respectively. *The fasciculation detection rate for each muscle group in ALS patients was significantly higher than non-ALS patients, *P* < 0.001

The distribution of fasciculation in ALS and non-ALS muscle groups is shown in Fig. 1B. Fasciculation was detected in 72.8% (1217/1672) muscle groups in ALS patients, which was significantly higher than that in muscle groups from non-ALS patients (18% [185/1026]) (*P* < 0.001). The detection rate of fasciculation for each muscle group was significantly higher in ALS patients than non-ALS patients (*P* < 0.001). No bulbar fasciculation was detected in non-ALS muscle groups (Fig. 2).

The fasciculation grade was 2 (0–3) in ALS muscle groups and 0 (0–0) in non-ALS muscle groups (*P* < 0.001). Also, the fasciculation grade for each muscle group was higher in ALS patients than non-ALS patients (Table 4).

**Table 4:** Comparison of fasciculation grades for each muscle group between ALS and non-ALS patients

Muscle groups	ALS		Non-ALS		Mean rank		Z value	***P*** value
	Number	Fasciculation grade	Number	Fasciculation grade	ALS	Non-ALS		
		(Median [IQR])		(Median [IQR])				
**Proximal flexors in upper limbs**	176	2 (1–4)	108	0 (0–0)	181.93	78.24	−10.978	**< 0.001**
**Proximal extensors in upper limbs**	176	2 (1–4)	108	0 (0–0)	181.26	79.33	−10.855	**< 0.001**
**Distal flexors in upper limbs**	176	2 (1–4)	108	0 (0–1)	178.60	83.68	−9.816	**< 0.001**
**Distal extensors in upper limbs**	176	2 (0–3)	108	0 (0–0.75)	170.49	96.89	−7.827	**< 0.001**
**Proximal flexors in lower limbs**	176	2 (1–4)	108	0 (0–0)	180.58	80.44	−10.513	**< 0.001**
**Proximal extensors in lower limbs**	176	2 (1–3)	108	0 (0–0)	177.66	85.21	−9.723	**< 0.001**
**Distal flexors in lower limbs**	176	2 (1–2)	108	0 (0–1)	172.50	93.62	−8.274	**< 0.001**
**Distal extensors in lower limbs**	176	1 (0–2)	108	0 (0–1)	167.47	101.81	−6.992	**< 0.001**
**Suprahyoid muscles**	88	0 (0–1)	54	0 (0–0)	81.01	56.00	−4.885	**< 0.001**
**Paravertebral muscles**	176	1 (0–2)	108	0 (0–0)	175.51	88.70	−9.890	**< 0.001**

*P-*values with significant differences are in bold type

**Fig. 3:**
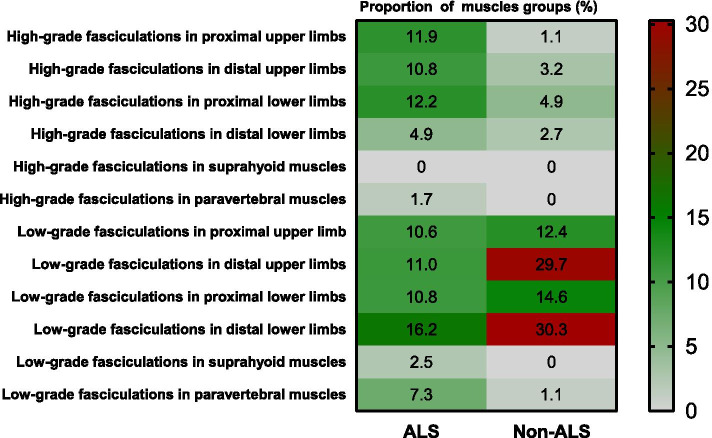
The distribution of high-grade and low-grade fasciculation in ALS and non-ALS muscle groups. The numbers in the figure represent the proportion of muscle groups with high-grade or low-grade fasciculation. For ALS patients, high-grade fasciculations were primarily distributed in the proximal muscle groups of lower limbs (12.2%) and upper limbs (11.9%), while low-grade fasciculations were primarily distributed in the distal muscle groups of lower limbs (16.2%) and upper limbs (11.0%). For non-ALS patients, most fasciculations were low-grade (88.1%) and primarily distributed in the distal muscle groups of lower limbs (30.3%) and upper limbs (29.7%)

The proportion of high-grade fasciculations was 41.5% (505/1217) in ALS muscle groups and 11.9% (22/185) in non-ALS muscle groups (*P* < 0.001). For ALS patients, high-grade fasciculations were primarily distributed in the proximal muscle groups of the lower limbs (12.2%) and upper limbs (11.9%). In comparison, low-grade fasciculations were mainly distributed in the distal muscle groups of the lower limbs (16.2%) and upper limbs (11.0%). For non-ALS patients, fasciculations were mostly low-grade (88.1%) and mainly distributed in the distal muscle groups of the lower limbs (30.3%) and of upper limbs (29.7%) (Fig. 3).

### Predictive value of fasciculation

**Fig. 4:**
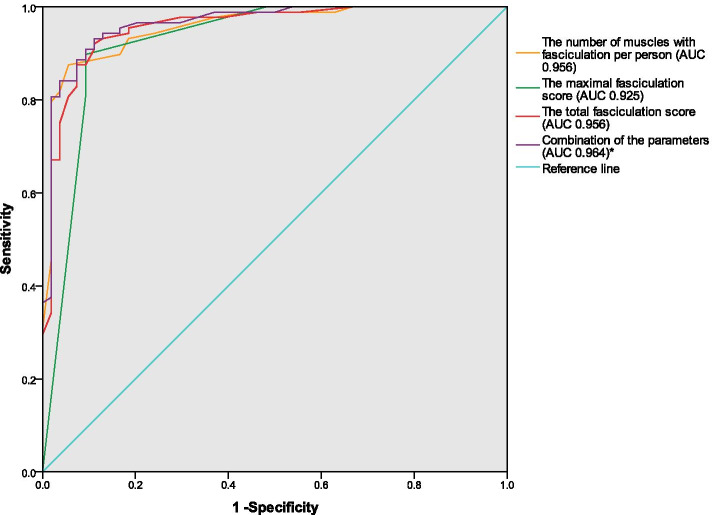
ROC analysis used to assess the discriminative potential of fasciculation detected by MUS between ALS and non-ALS patients. * Combination of the number of muscles with fasciculation per person, the maximum fasciculation score and the total fasciculation score

ROC analysis demonstrated that for the number of muscles with fasciculation per person, the area under the curve (AUC) was 0.956 (95% confidence interval [CI] 0.923–0.988), with the sensitivity and specificity for the diagnosis of ALS at 87.5 and 94.4% (cut-off value 8.5), respectively. For the maximum fasciculation score, the AUC was 0.925 (95% CI 0.873–0.977), with the sensitivity and specificity for the diagnosis of ALS 89.8 and 90.7% (cut-off value 2.5), respectively. For the total fasciculation score, the AUC was 0.956 (95% CI 0.922–0.990), with the sensitivity and specificity for the diagnosis of ALS 92.0 and 88.9% (cut-off value 11.5), respectively. When the above three parameters were combined for ROC analysis, the AUC improved to 0.964 (95% CI 0.934–0.993) (Fig. 4).

## Discussion

Fasciculation is a characteristic manifestation of ALS patients. However, it also can be detected in healthy people, those with cramp-fasciculation syndrome, in certain metabolic diseases, and other neuromuscular diseases [16, 17]. We found that compared with non-ALS patients, including patients in the present study with peripheral neuropathy and cervical spondylopathy or lumbar spondylopathy, a significantly higher detection rate and higher grade of fasciculation were observed in ALS patients when examined with MUS. Moreover, the fasciculations in ALS patients always were diffuse and persistent (grades 3 and 4 fasciculation). The fasciculation observed in non-ALS patients was mostly focal or multifocal and intermittent (grades 1 and 2 fasciculation). Moreover, ROC analysis revealed that assessment of fasciculation was of great value for the diagnosis of ALS. These results are consistent with previous studies. Tsuji et al. reported that fasciculations were widely distributed in ALS patients and locally distributed in non-ALS patients [9]. The study by Johansson et al. has shown that the main type of fasciculation in ALS is continuous (45.4%) [14]. Wang et al. found that the number of muscles with fasciculation in ALS patients was significantly greater than patients with peripheral neuropathy or cervical spondylopathy and healthy participants [18]. Fasciculation was not only more common in ALS patients, but also associated with disease burden and activity. Avidan et al. [10] reported that high fasciculation frequency at the biceps brachii and brachialis muscles, where detection rate was the highest under MUS, was associated with less impairment at time of examination, and a more active disease with a more rapid progression. This finding showed that fasciculation frequency might provide prognostic information.

The non-ALS diseases we studied included MMN, CIDP, CMT, as well as cervical spondylosis or lumbar spondylosis, which could involve the conduction pathways of lower motor neurons (e.g., nerve roots or peripheral nerves). Focal demyelination of peripheral motor axons can generate increased axon excitability and ectopic discharge, which likely is an explanation for the occurrence of fasciculation in non-ALS patients [19]. Also, axon excitability studies have confirmed that the Na+/K+ pump at the MMN injury site is blocked, resulting in depolarization and hyperpolarization areas surrounding the conduction block site, which reduces the stability of the axonal membrane and causes abnormal excitability potentials [20, 21].

In previous studies, fasciculation has been observed in both upper and lower limbs in patients with cervical spondylotic myelopathy, indicating the presence of an incidental complication of lumbar spondylopathy or that fasciculation in the lower limbs probably originated from upper motor neurons [22]. Interestingly, the maximal fasciculation score in a small proportion of non-ALS patients (9.3%) was 4 in this study. They were all patients with peripheral neuropathy, but none were patients with cervical or lumbar spondylopathy, suggesting possible increased nerve hyperexcitability in peripheral neuropathy.

In the present study, the high-grade fasciculations of ALS patients were primarily distributed in the proximal limbs, while low-grade fasciculations were mainly distributed in the distal limbs. The fasciculations of non-ALS patients were mainly distributed in the distal limbs, which was consistent with the research results of Johansson et al. and Higashihara et al. [14, 23]. Fasciculation in ALS, which is thought to originate from upper and lower motor neurons, is associated with hyperexcitation of the motor cortex and axons [24]. Early in the progression of ALS, when fasciculation is the main symptom, and no muscle weakness is observed, processes related to upper motor neurons dominate. Later, with the progressive dysfunction of lower motor neurons, gradual muscle weakness occurs, complex and highly unstable motor units form, and peripheral motor axons initiate the production of ectopic activities [20]. However, for the non-ALS patients in our study, most fasciculations originated from only lower motor neurons and were partially related to nerve length dependence. These might be the reasons for the different distribution of fasciculation in ALS and non-ALS patients [25]. No fasciculation was detected in suprahyoid muscles for non-ALS patients in this study. Therefore, fasciculation in this area was highly specific for distinguishing ALS from non-ALS diseases.

Ultrasound was first applied in the observation of fasciculation by Walker et al. [26] in 1990. In addition to noninvasiveness, easy operation and higher sensitivity, MUS have showed other benefits with the clinical application. Firstly, it takes at least 70–90 s to record fasciculation. However, the time can be shortened to 60 s due to the significantly larger observation area using MUS, which greatly reduces the examination time for each patient [11]. Besides, the lower cost of MUS makes it more acceptable, which reduces the economic burden of patients. Moreover, as for the follow-up of patients, the interval between EMG examinations is usually at least 3 months, while MUS can be performed at any time without such limitation. MUS provides a new method for ALS diagnosis. According to Awaji criteria, the discovery of fasciculation under MUS revises 6% of the possible diagnosis to probable diagnosis [14].

When detecting fasciculations by MUS, we used muscle groups instead of individual muscles as the observation unit, which allowed a wide range of muscles to be detected simultaneously in one region, which saved time. Also, this method was easy to perform. Furthermore, we classified the intensity of fasciculation according to firing frequency and site number in the muscles involved during the examination, which quantified the severity of fasciculation and provided a more intuitive evaluation. However, our study also presented several limitations. First, the study was cross-sectional and did not assess fasciculation changes longitudinally. Second, we did not comprehensively analyse the fasciculation under MUS in combination with other parameters, such as EMG assessment and muscle thickness. Finally, we did not compare the fasciculation that occurred in each of the different diseases with ALS due to the small number of non-ALA patients with each type of disease.

In summary, ultrasound technology can detect fasciculation effectively. The detection rate and grade of fasciculation in ALS were significantly higher than that seen with non-ALS diseases. Fasciculations were primarily distributed in the proximal limbs in ALS patients. Fasciculation that occurred in the suprahyoid muscles exhibited high specificity for distinguishing ALS from non-ALS diseases. In the future, the sample size could be increased to explore the difference of fasciculation between ALS and specific non-ALS diseases. We conclude that these findings will facilitate the clinical diagnosis of these diseases.

## Supplementary Information

**Additional file 1.**

**Additional file 2.**

## Data Availability

The datasets generated and/or analysed during the current study are not publicly available due to privacy or ethical restrictions. But are available from the corresponding author on reasonable request.

## Abbreviations

ALS: Amyotrophic lateral sclerosis
MUS: Muscle ultrasonography
EMG: Electromyography
PN: Peripheral neuropathy
BMI: Body mass index
ALSFRS-R: ALS Functional Rating Scale-Revised
MRC: Medical Research Council
MMN: Multifocal motor neuropathy
CIDP: Chronic inflammatory demyelinating polyneuropathy
CMT: Charcot-Marie-Tooth
AUC: Area under the curve
CI: Confidence interval
