# Supplementary material for: Fasciculation differences between ALS and non-ALS patients: an ultrasound study
Source: BMC Neurol. 2021 Nov 10;21:441. doi: 10.1186/s12883-021-02473-5 (PMC8579676; doi:10.1186/s12883-021-02473-5)
Supplement: Supplementary file 1 — Additional file 1. [file 12883_2021_2473_MOESM1_ESM.docx]

**The details on patients with cervical radiculopathy**

| Patient | Affected root level | Total fasciculation score | The maximum fasciculation score | The distribution of fasciculation |
| --- | --- | --- | --- | --- |
| 1 | C2-C5 | 0 | 0 |  |
| 2 | C6-C7 | 0 | 0 |  |
| 3 | C4-C6 | 0 | 0 |  |
| 4 | C5-C6 | 0 | 0 |  |
| 5 | C5-C7 | 2 | 1 | distal muscle groups of lower limbs |
| 6 | C3-C7 | 2 | 2 | distal muscle groups of upper limbs |
| 7 | C5-C7 | 3 | 2 | distal muscle groups of upper limbs |
| 8 | C6-C7 | 5 | 2 | distal muscle groups of upper limbs, distal and proximal muscle groups of lower limbs |
| 9 | C5-C7 | 8 | 2 | distal and proximal muscle groups of upper limbs |
